# Plant-based meat substitutes on the German market: a characterization based on declared nutrient contents, Nutri-Score, organic and vegan labeling, and use of iodized salt

**DOI:** 10.3389/fnut.2026.1730844

**Published:** 2026-04-09

**Authors:** Corinna Gréa, Melanie Turke, Laura Busl, Stefan Storcksdieck genannt Bonsmann

**Affiliations:** Department of Nutritional Behaviour, Max Rubner-Institut (MRI) - Federal Research Institute of Nutrition and Food, Karlsruhe, Germany

**Keywords:** German product monitoring, iodized salt, meat substitutes, Nutri-Score label, nutrition labeling, plant-based meat alternative, vegan, vegetarian

## Abstract

A varied, predominantly plant-based diet is recommended for both individual and planetary health. In line with growing interest in limiting meat consumption, the market for plant-based meat substitutes (PBMS) has grown substantially in recent years, especially in Germany. Constant monitoring of this market is warranted to understand key nutritional and sustainability characteristics of PBMS. Using food labeling data of 964 PBMS recorded within the German monitoring of packaged food in 2024, we report the energy and nutrient content per 100 g, use of iodized salt, the presence of Nutri-Score as well as organic and vegan labeling overall and by (sub)category. Median energy/nutrient contents in PBMS were 224 kcal, 12.0 g fat, 13.3 g protein, and 1.60 g salt. At category level, meat substitutes showed lower median fat and salt contents than sausage substitutes, which was echoed in more favorable Nutri-Score ratings, where provided (19% of all products). At subcategory level, raw sausages had the highest median contents of energy, sugars, protein and one of the highest medians in salt content. About one third of all products was labeled as organic, more than 85% as vegan. Less than 6% of the PBMS contained iodized salt. The results show a high diversity of PBMS in the German market, with slightly more favorable nutritional composition of meat substitutes compared to sausage substitutes. Given the heterogeneity of products, more widespread Nutri-Score labeling could help guide consumers in their choices. PBMS manufacturers should consider shifting to iodized salt in their product formulations.

## 1 Introduction

There is growing consensus that more sustainable dietary habits are needed for individual and environmental health (1), one decisive factor being the reduction of meat consumption (2). Plant-based meat substitutes (PBMS) may serve as a versatile food option toward this goal without requiring consumers to fundamentally change their dietary behavior. These products are designed to be similar to traditional meat and sausage products in terms of function, sensory properties, appearance, and names (3).

In Germany, the proportion of people who consume vegetarian or vegan alternatives to animal products, including PBMS, on a daily basis increased from 5% in 2020 to 10% in 2024 (4). At the same time, PBMS production has more than doubled, from 60,400 tons in 2019 to 121,600 tons in 2023, and continues to rise (5). Between September 2023 and August 2024, about a third of all German households purchased PBMS at least once, and the sales volume averaged 3.7 kg per household (6). The growing consumption of PBMS and the increasing number of newly launched products show that PBMS are becoming ever more relevant in everyday nutrition (7).

Given the highly variable composition of PBMS, in some cases resulting in high levels of salt or saturated fat (8), regular monitoring of the product offer can identify compositional issues and guide reformulation efforts. In Germany, this is taken care of by the national product monitoring of packaged food, which was established as part of the “National Reduction and Innovation Strategy for Sugar, Fats, and Salt in Processed Foods” (hereafter NRI) (9). The monitoring focuses on product groups of known or potential relevance to sugar, fat, and salt intake, and aims to cover the respective market as completely as possible. A special focus of the NRI is on food groups targeted at children (10). The gathered packaging information includes the declared nutrient contents, ingredient lists, and further items such as Front-of-Pack Labeling (FoPL). For context, FoPL is voluntary in the EU as per current regulation. The Nutri-Score, a color-coded, evaluative FoPL, has been adopted in Germany and is intended to facilitate comparisons between products within a food category (11). Nutri-Score grades products from A (dark green) to E (dark orange) according to their nutritional composition, counting fiber, protein, fruit and vegetables as positive components and saturated fatty acids (SFA), sugar and salt as negative components (12).

Besides the nutritional value of food products, consumers may also be sensitive to further labeling information such as organic origin or purely vegan ingredients (13). Based on the latest data from the national product monitoring, this brief research report provides insights into the labeled energy and nutrient composition of PBMS on the German market, the use of the Nutri-Score as well as vegan and organic labeling on these products. Against the backdrop of declining iodine intake in Germany (14), we further investigated fortification with iodine (in the form of iodized salt) within this product group. As natural sources of iodine in the diet are mainly animal-based products, fortification among plant-based alternatives is particularly important to support adequate iodine intake.

## 2 Methods

For the present analysis, data on PBMS on the German market, collected in 2024 (6) as part of the German national product monitoring of packaged food [see (10) for details], was used. In each survey year, product data are collected from August to December. Only packaged food products available in the German retail market with a European Article Number (EAN) or Global Trade Item Number (GTIN) are included. To cover the products available on the market as completely as possible, data collection follows a stepwise process including manual online research on original manufacturers’ websites, enquiries with manufacturers as well as on-site research in grocery stores. Data collection focuses on mandatory nutrition declaration of energy, fat, SFA, protein, carbohydrates (CHO), sugars, and salt as the so called Big 7, ingredient list, and further packaging information such as Nutri-Score or organic production.

Data were managed with a customized branded food module within the FoodCASE software, version 8.8.0 (Premotec GmbH, Winterthur, Switzerland).

All calculations were performed using R (version 4.5.0) and RStudio (version 2025.05.0 + 496). Values for energy were rounded to no decimal, fat, SFA, CHO, sugars, protein, and all percentages were rounded to one decimal, salt values to two decimals.

### 2.1 Definition of PBMS and inclusion criteria

Plant-based meat substitutes are made from vegetarian or vegan ingredients, with a meat-like texture, design, or taste. For such products to be included as PBMS in the German product monitoring of packaged food, the name, the labeling, or the product description had to contain terms of an originally meat-based product such as schnitzel, (ham)burger, or sausage. PBMS without a textual reference to meat were only included if the design of the packaging or the product alluded to a meat product. Products not designed to imitate meat products, such as plain tofu blocks and dry products (e.g., granulated soy for reconstitution), were excluded.

According to the intended use and consumption occasions, PBMS were subdivided into the two categories of meat substitutes and sausage substitutes, and within those categories further divided into the following subcategories:

#### 2.1.1 Subcategories of meat substitutes

- Minced products: e.g., burger patties, meatball substitutes.
- Pan-cooked products: diced or chunky meat substitutes, vegan or vegetarian filet or steak.
- Breaded products: e.g., nuggets, filled and unfilled schnitzel.
- Other meat substitutes: products that could not be assigned to any specific meat substitutes subcategory (e.g., delicatessen salad with meat substitutes, roast substitutes).
- Meat substitutes targeted at children:

Any kind of meat substitute is assigned to this category if at least one of the following criteria is fulfilled:

- Product name includes “child(ren)” or “kids” or appeals directly to children.
- Packaging is attractively designed for children.
- Food product itself or its components are designed for children.
- Packaging includes information aimed at parents or children.

Assignment as product targeted at children overrides further assignment to any of the other subcategories.

#### 2.1.2 Subcategories of sausage substitutes

- Raw sausages: salamis and further substitutes for raw sausages.
- Spreads: spreadable sausage substitutes such as liver sausage spread.
- Cooked sausages and slices: vegan and vegetarian cold cuts, substitutes for boiled, cooked, and fried sausages, wiener.
- Ham and bacon: substitutes for cooked ham and bacon including bacon cubes.
- Sausage substitutes targeted at children:

Any kind of sausage substitute is assigned to this category if at least one of the criteria for products targeted at children is fulfilled (See criteria description above for subcategory *Meat substitutes targeted at children*).

### 2.2 Labeling parameters examined

#### 2.2.1 Energy and nutrient content

Descriptive statistics, including median, mean, minimum and maximum for energy, fat, SFA, CHO, sugars, protein, and salt in g/100 g product, are shown for the overall sample, the subsample targeted at children, and the categories and subcategories (Table 1).

**TABLE 1:** Energy and nutrient contents in PBMS for the overall sample, products targeted at children, categories, and subcategories.

(Sub)categories of PBMS	*n*	Content per 100 g of product													
		Energy in kcal		Fat in g		SFA in g		CHO in g		Sugars in g		Protein in g		Salt in g	
		Median/ mean	Min/ max	Median/ mean	Min/ max	Median/ mean	Min/ max	Median/ mean	Min/ max	Median/ mean	Min/ max	Median/ mean	Min/ max	Median/ mean	Min/ max
PBMS total	964	224/217	50/466	12.0/12.8	0.1/49.0	1.3/2.3	0.0/23.0	7.0/9.2	0.3/34.3	1.6/2.0	0.0/14.0	13.3/14.8	0.6/42.3	1.60/1.70	0.03/4.16
PBMS targeted at children	58	221/224	90/339	11.1/13.5	4.3/33.0	1.4/2.9	0.4/23.0	7.4/8.9	1.0/28.5	1.9/2.1	0.0/8.7	12.0/15.2	2.7/30.6	1.60/1.73	0.70/3.30
Meat substitutes	506	219/216	50/466	11.0/11.5	0.1/49.0	1.2/1.8	0.0/16.0	9.0/11.8	0.9/34.3	1.5/2.0	0.0/14.0	14.0/14.3	0.6/42.3	1.35/1.41	0.03/3.90
Minced products	185	212/206	78/336	10.0/10.9	0.5/23.2	1.2/2.2	0.1/16.0	8.3/11.6	1.0/32.0	1.8/2.1	0.0/14.0	13.1/13.5	1.6/34.9	1.30/1.34	0.20/2.34
Pan-cooked products	108	194/190	50/350	9.0/9.7	0.1/29.0	1.0/1.3	0.0/8.8	4.9/6.0	0.9/31.0	1.0/1.4	0.0/9.8	18.0/17.7	1.5/28.6	1.40/1.50	0.03/3.20
Breaded products	123	241/241	106/328	12.0/12.5	3.6/22.0	1.3/1.8	0.2/10.9	19.6/19.1	2.8/34.3	1.2/1.8	0.0/9.4	11.0/11.2	2.9/25.0	1.30/1.33	0.20/2.10
Other meat substitutes	58	230/234	50/466	12.0/15.1	0.1/49.0	1.4/1.8	0.1/9.6	7.2/7.7	1.0/19.0	2.1/3.4	0.1/13.0	15.0/15.2	0.6/42.3	1.60/1.65	0.03/3.90
Meat substitutes targeted at children	32	220/228	141/337	9.6/11.7	4.5/32.0	1.1/1.7	0.4/6.3	9.9/11.9	3.9/28.5	1.7/2.3	0.4/8.7	14.0/17.2	4.2/30.0	1.35/1.44	0.70/2.10
Sausage substitutes	458	229/220	60/409	14.0/14.2	0.5/38.0	1.5/2.8	0.1/23.0	5.7/6.3	0.3/18.3	1.6/1.9	0.0/9.0	13.0/15.4	1.0/38.0	1.90/2.01	0.90/4.16
Raw sausages	48	252/266	131/358	12.7/15.1	7.7/31.4	2.2/3.5	0.3/19.7	8.3/8.5	2.7/17.1	2.7/3.3	0.0/9.0	26.5/22.3	5.0/38.0	2.50/2.64	1.20/4.00
Spreads	54	251/247	66/388	21.0/20.1	2.5/36.0	2.1/5.4	0.2/22.8	8.9/9.7	4.2/17.6	1.8/2.1	0.6/6.7	5.2/5.4	2.5/13.1	1.70/1.81	1.30/2.70
Cooked sausages and slices	312	220/210	66/409	13.0/13.3	2.0/38.0	1.3/2.2	0.2/14.5	5.1/5.5	0.3/18.3	1.5/1.6	0.0/4.4	15.1/16.1	1.9/36.0	1.80/1.91	0.90/4.10
Ham and bacon	18	208/183	60/300	6.0/8.1	0.5/20.0	1.2/1.3	0.1/4.9	7.3/6.5	1.3/13.0	1.8/2.6	0.6/5.6	18.3/20.1	1.0/36.0	2.55/2.52	1.10/4.16
Sausage substitutes targeted at children	26	226/218	90/339	16.5/15.8	4.3/33.0	2.2/4.3	0.4/23.0	4.2/5.2	1.0/11.7	2.0/1.9	0.0/3.5	8.8/12.8	2.7/30.6	2.00/2.08	1.10/3.30

CHO, carbohydrates; Max, maximum; Min, minimum; PBMS, plant-based meat substitutes; SFA, saturated fatty acids.

#### 2.2.2 Provision of Nutri-Score

The provision of the Nutri-Score was assessed on the level of both the overall sample and the category.

Packaging of the included PBMS was checked for any Nutri-Score labeling and, if present, the actual score (A–E) recorded. In case of unavailable packaging, manufacturers’ websites were checked for further information on the use of Nutri-Score. Nutri-Score classes A–E are shown as percentage share.

#### 2.2.3 Organic and vegan labeling

To assess the share of organic products within the overall sample and at category level, packaging was checked for the presence of organic labeling. Products were considered as organic if the official organic label of the European Union or an official German national organic label, such as “Bioland,” “Demeter,” or “Naturland,” was present on the packaging and/or the word “bio” or “eco” was recognizable in the product or sales name. In cases where packaging information was only partly available (e.g., only front of pack), manufacturers’ websites were checked for further information, including whether producers were recognized as 100% organic.

The proportion of vegan products was investigated for the overall sample and at category level. Products were considered as vegan if either a national vegan label was present on the packaging and/or if the term “vegan” was mentioned in the product or sales name, or if terms such as 100% plant-based, purely vegetable, or vegetal were present on the packaging. If no packaging was available, the manufacturer’s website was checked for further information or a direct inquiry performed.

#### 2.2.4 Use of iodized salt

Given the mandatory labeling of iodized salt as a food ingredient in the EU, its use was computed as the share of all products with an available ingredients list and containing salt [see (15) for details], both for the overall sample and at category level.

## 3 Results

The comprehensive search for PBMS on the German market in 2024 yielded a total of 964 products, of which 6.0% (*n* = 58) were classified as PBMS targeted at children. Both the overall sample and PBMS targeted at children contained slightly more meat substitutes than sausage substitutes. Cooked sausages and slices formed the largest subcategory, accounting for about one-third of all products surveyed.

### 3.1 Energy and nutrient contents

For the sake of simplicity, the description of the energy and nutrient contents (see Table 1) focus on the median and its differences between categories and subcategories.

For the overall sample, median energy content was 224 kcal/100 g, median protein content 13.3/100 g, and median salt content 1.60/100 g. The corresponding medians for PBMS targeted at children differed only slightly.

On the category level, sausage substitutes tended to have higher median contents per 100 g for energy (229 kcal), fat (14.0 g), SFA (1.5 g), and salt (1.90 g) than meat substitutes. More distinct differences in ranges and medians were observed for CHO. Median protein and sugar contents were similar between both categories and the overall sample, and both categories exhibited wide ranges in energy and nutrient contents.

At subcategory level, raw sausages had (one of) the highest median contents per 100 g for energy (252 kcal), SFA (2.2 g), sugars (2.7 g), and protein (26.5 g), and the second highest median salt content (2.50 g, only surpassed by ham and bacon with 2.55 g). Spreads showed the highest median fat content (21.0/100 g), and breaded products the highest median CHO content (19.6/100 g). The lowest median contents for energy, SFA, and sugars were seen in pan-cooked products, whereas spreads showed the lowest median protein content (5.2/100 g). Minced and breaded products, both assigned to meat substitutes, showed the lowest median salt content (1.30/100 g). Most subcategories showed wide ranges in energy and nutrient contents.

Since the subcategories targeted at children lack the specificity of the other subcategories in terms of grouping together like products, the comparative analysis of their energy and nutrient contents is omitted.

### 3.2 Provision of Nutri-Score

About one fifth (19.3%) of all PBMS showed a Nutri-Score on their product packaging or the manufacturer’s webpage (data not shown). Of these, the majority (61.8%) was labeled with Nutri-Score A or B. Among the 58 products targeted at children, nine had a Nutri-Score, all either A or B.

**FIGURE 1:**
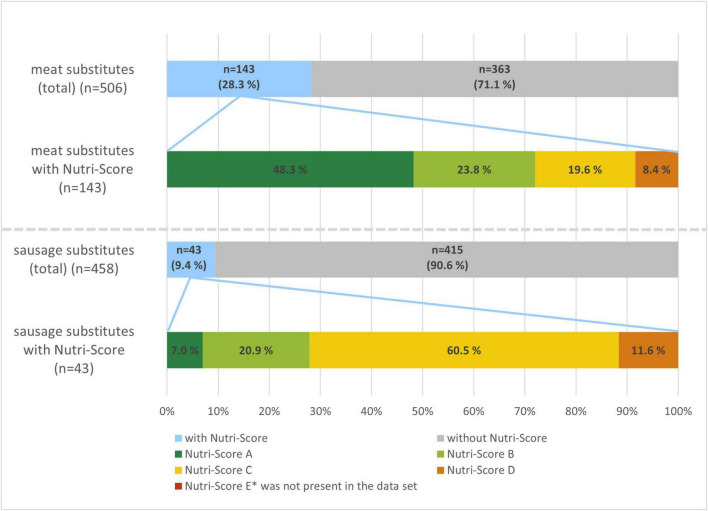
Use of Nutri-Score and corresponding classes within meat substitutes (upper panel) and sausage substitutes (lower panel).

At category level, the percentage of PBMS with a Nutri-Score was three times higher among meat substitutes than among sausage substitutes (28.3% vs. 9.4%), see Figure 1.

While no product was labeled with Nutri-Score E, the distribution of Nutri-Score classes differed substantially between meat and sausage substitutes. Almost three quarters (72.1%) of the 143 labeled meat substitutes scored A or B, whereas the same share of the 43 labeled sausage substitutes scored C or D (Figure 1).

### 3.3 Organic and vegan products

**FIGURE 2:**
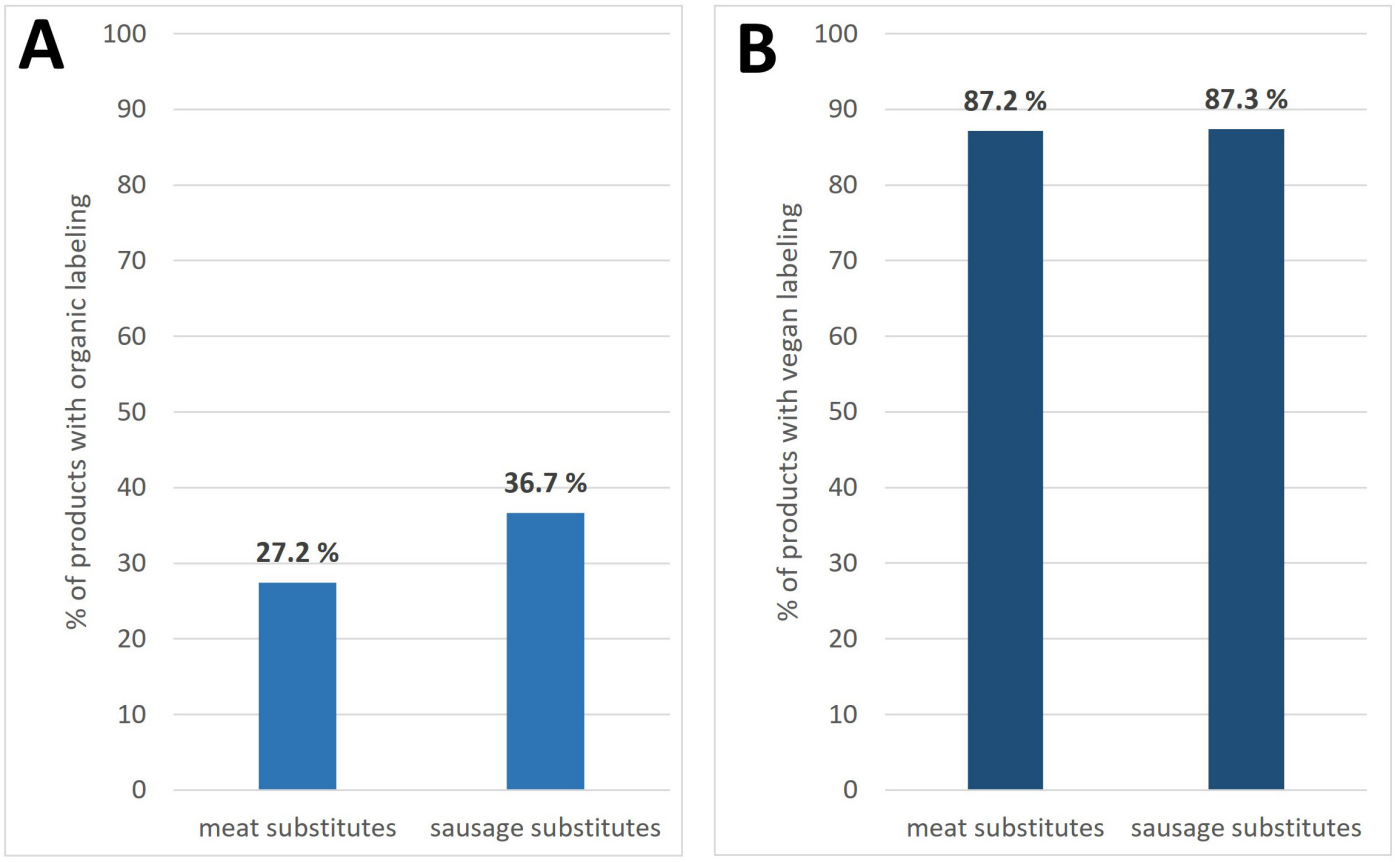
Percentage of products with organic **(A)** and vegan **(B)** labeling in meat substitutes and sausage substitutes.

Of the overall sample, 31.9% of products were from organic production (data not shown). The share of organic products was slightly higher among sausage substitutes than meat substitutes (36.7% vs. 27.5%) (see Figure 2A). Around 87% of the overall sample was labeled as vegan, with no difference between categories (see Figure 2B).

### 3.4 Use of iodized salt

Iodized salt was used in 5.7% (*n* = 54) of all PBMS with salt in the ingredient list (*n* = 947), the shares being nearly the same for meat substitutes and sausage substitutes (5.5% vs. 6.0%) (data not shown).

## 4 Discussion

Using a comprehensive and recent data set of PBMS available on the German market, this brief research report gives a high-resolution account of energy and nutrient contents (Big 7) of major PBMS categories and subcategories. Furthermore, it provides insights into this product group as regards the use of iodized salt as an ingredient as well as the spread of Nutri-Score, organic, and vegan labeling.

### 4.1 Energy and nutrient contents

With the proliferation of PBMS, several studies have investigated their energy and nutrient composition. Owing to the inherent role of PBMS as a potential replacement for meat in the diet, protein content is a focus of discussion (8, 16). Other studies from Germany, all with substantially smaller sample sizes, reported mean protein contents of 14–21/100 g (17), and 5–21/100 g (18), depending on the respective subcategories. Our study also found large ranges of mean protein contents between subcategories, with values of 5/100 g in spreads and 22/100 g in raw sausages. On the category level, similar results regarding protein content of PBMS as in our study have been reported in studies from other European countries, e.g., Italy or Denmark, in which median protein content was about 14 g (16, 19, 20). In contrast, our data showed higher values for fat and salt than these European studies. The differences in energy and nutrient values may be explained by different national product formulations and food offer resulting in different sample compositions. For example, our sample included a high number of sausage substitutes, which had higher median values for fat and salt than meat substitutes. Due to the diverse national market situations and food cultures, direct comparisons on (sub)category level are even more limited. For example, no previous study outside of Germany specifically included spreads such as substitutes for liver sausage or pork paté, which in our analysis showed the highest median fat content. Regarding a comparison of the protein content on the subcategory level, we could identify only one study, based on PBMS on the Spanish market, which also observed higher protein contents in sausage substitutes than in other substitutes (burgers, meat balls, and nuggets) (21). Within subcategories, our study revealed a particularly high salt content of raw sausages, which may be caused by a desired similarity to the salty flavors typical for raw meat sausages.

For both meat substitutes and sausage substitutes, we differentiated products targeted at children as a separate subcategory. Those subcategories contained a great variety of different products, and their overall share of the total sample was small. However, in line with a growing market and public health interest in products targeted at children, future development in this area should be monitored. In 2023, the World Health Organization (WHO) published an updated nutrient profile model for restricting marketing of unhealthy foods to children (22). Within the category “savory plant-based foods/meat analogues,” nutrient/ingredient thresholds were set as follows: A maximum level of 17 g fat and 0.5 g sodium per 100 g as well as the absence of added sugars and non-sugar sweeteners. A corresponding assessment within the German product monitoring showed that only 7 out of 58 PBMS targeted at children met these criteria (23). Since exceeding the sodium threshold (see Table 1) was the main reason for the non-fulfillment of the WHO nutrient profile, salt reduction remains a major topic for product development and reformulation of PBMS.

As regards the nutritional comparison of PBMS with their meat counterparts, a recent review of studies from different countries reported a more favorable nutritional profile of PBMS (8). For Germany, a detailed comparative analysis by us with meat and sausage products based on monitoring data from an earlier survey showed that most of the PBMS subcategories had significantly lower contents of fat and SFA but higher contents of CHO and sugars than corresponding meat subcategories (24). Our results are similar to a more recent study from Germany, which compared the declared nutritional values of 298 PBMS with 294 meat counterparts for which data where mainly gathered from the German Nutrient Database (18). Overall, our findings are in line with the general observation that PBMS differ greatly in terms of energy and nutrient composition, even within the same product (sub)category (8). Major underlying reasons for the observed variety are the range of raw materials used, such as different pulses, legumes, or isolated plant proteins, and the substantial compositional freedom. Consequently, consumers are faced with a wide spectrum of products and nutrient profiles, which provides choice but may also be challenging as regards informed decision-making. FoPL that empowers consumers to identify nutritionally favorable options at a glance could help alleviate the challenge.

### 4.2 Provision of the Nutri-Score

Seven European countries (Belgium, France, Germany, Luxemburg, Spain, Switzerland, Netherlands) have adopted the Nutri-Score as their national voluntary FoPL of choice. Within those countries, the proportion of labeled products varies. Up-to-date information of Nutri-Score coverage on the German market is scarce, let alone for PBMS. A market check by German consumer organizations in 2022 for the six product groups breads and rolls, pizzas, milk and dairy drinks, plant-based drinks, and cereals showed that about 40% of the 1,451 products inspected bore the Nutri-Score, with the highest share among pizzas (70%) and the lowest among milk and milk drinks and cereals (each 28%) (25). In comparison, our study showed about 20% Nutri-Score labeling for PBMS in 2024. Further food product groups covered in 2024 by the national product monitoring showed a share of 7% for soft drinks, 12% for pastries, and 18% for cold sauces (26).

We also noted substantial differences in the Nutri-Score labeling between the two categories, both in availability and in actual scoring. Among sausage substitutes, where only 10% of all products carried a Nutri-Score, scores were mostly C or D. In contrast, some 30% of meat substitutes bore the Nutri-Score, and these products mostly scored A or B. As the aim of this paper was to describe the market situation for PBMS in Germany from a consumer perspective, we did not calculate the Nutri-Score for products where it was not used. However, a recent study from Germany (18) computed the Nutri-Score for the entire sample of PBMS (*n* = 298) and also noted that meat substitutes had higher proportions of favorable ratings (A or B) than sausage substitutes. In that study, 27% of meat substitutes were rated A or B, compared to 4% of the sausage substitutes.

In 2023, the Nutri-Score algorithm underwent a revision to improve the discrimination between products with high and low contents of unfavorable nutrients (27). This includes a stricter evaluation of salt and sugar contents as well as a higher threshold for protein content. A recent Dutch study showed that the adaption resulted in a shift to a less favorable Nutri-Score for some PBMS. The percentage of products labeled with A or B decreased by 5% for cold cuts alternatives, and 15% for meat alternatives (28). As the new algorithm was introduced in January 2024, with a 24-month transition period in Germany, it is likely that our sample contains a mix of Nutri-Score labeling based on the old or the new algorithm.

When interpreting the Nutri-Score it is important to consider its intended application as FoPL to guide consumer choices toward nutritionally more favorable options within a food category. The underlying algorithm trades off selected positive and negative nutrient/ingredient dimensions (e.g., salt content as a negative, fruit/vegetable content as a positive). Aspects like fortification or the use of additives are not considered. As such, the Nutri-Score is not designed to comprehensively assess the healthiness of a product.

### 4.3 Organic and vegan products

Similar to studies by others (16, 29), the vast majority of PBMS in our survey were labeled as vegan. The share of vegetarian PBMS on the German market, which typically contain milk or egg derivatives, has declined in recent years in favor of vegan products (own unpublished data).

With about 32%, the share of products labeled as organic is similar to an earlier study from Germany (17) and fits with a current market observation showing a significantly higher share of organic products among plant-based alternatives than in longstanding food groups such as eggs or milk (30). The high proportion of organic and vegan labeling may be related to the fact that vegetarians and vegans, who tend to be health- and environment-conscious, are the primary and still growing consumer groups for plant-based alternatives (31). This notwithstanding, PBMS are also increasingly designed to appeal to omnivores as an additional consumer group (32, 33).

### 4.4 Use of iodized salt

The very low share of PBMS with iodized salt (around 6%) within our sample should be closely monitored as iodine supply in the German population is already on the decline (14, 34, 35). Especially with a fully or predominantly vegetarian or vegan diet, fortification with iodine is needed since an inadequate intake can only partially or not at all be compensated with natural sources such as marine fish, milk, and eggs (35, 36).

### 4.5 Strengths and limitations

Key strengths of the present report derive from its comprehensive data set, with product information gathered systematically from manufacturer websites and visits to retail stores. The data set comprises food products of both brand manufacturers and discount brands, and it allows for investigating a variety of distinct subcategories. The present study can be seen as covering the German market and thus differs from other studies in the field, which are often limited by one or more of the following: far fewer products, less granularity, or the use of external databases for which it may not be entirely clear how current they are vis-á-vis the actual market situation.

The nutritional content assessment provided is based on mandatory nutrition labeling on packaged products, which is limited both in detail (only Big 7) and precision [EU regulations permit a discrepancy of up to 20% from actual content as determined by chemical analysis (24, 37)]. However, the vast number of products on the German market, which is the largest retail market for PBMS in Europe (16), would not be feasible to analyze chemically for the purpose of a regular and comprehensive monitoring.

A further limitation lies in the fact that the declared nutrient contents are insufficient to make a full assessment of the nutritional equivalence of PBMS to one another or to their animal counterparts. However, it is important to acknowledge that the substantial compositional variability of PBMS makes such an assessment a daunting task. Current research in this area shows (8) that protein biological value and nutrient bioavailability may differ significantly between products. A full qualitative analysis of the ingredient list and quantitative investigation of the micro- and macronutrient composition of individual products, e.g., using estimation algorithms, are beyond the scope of this paper, but could be examined further in the future.

### 4.6 Directions for future research

The increasing relevance of PBMS in people’s diet requires careful assessment of their nutritional quality. In this regard, future research should explore the reformulation potential hidden in the compositional variability of this product group, seeking to minimize the contents of sugars, SFA, and salt while maintaining food safety (microbiologically and chemically). Since the protein content of PBMS is not necessarily inferior to their meat counterparts, attention should be given to achieving similar protein quality. This could be assessed in *in vitro* bioavailability trials, which may also serve to study adequate micronutrient provision, including from fortification. Of note, products may not be exchanged on a one-to-one basis and many consumers today combine meat and PBMS in their diet (4). However, continuous product monitoring and dietary surveys are needed to observe the contribution that PBMS make to people’s diets and what this means for nutrition and health. For example, an updated comparison of PBMS with their meat and sausage counterparts based on future product monitoring data or the calculation of the Nutri-Score for unlabeled products could be considered.

## 5 Conclusion

The market of PBMS in Germany is characterized by substantial variability in terms of product and nutritional composition, as judged by the mandatory labeling information. Compared to meat substitutes, sausage substitutes tend to be less nutritionally favorable owing to their higher average fat and salt contents. Given the diversity of products and the continuing growth and development of the market of PBMS in Germany, a higher share of products using the Nutri-Score could be helpful to guide consumers in their choices. Manufacturers should increase the use of iodized salt, especially where PBMS are used to replace animal-based products that are important dietary sources of iodine. Continued monitoring of PBMS is warranted to track the further evolution of this highly variable product group.

## Data Availability

The raw data supporting the conclusions of this article will be made available by the authors, without undue reservation.
